# Complete remission after sintilimab combined with chemoradiotherapy in double primary head and neck carcinoma: case report

**DOI:** 10.3389/fonc.2024.1397877

**Published:** 2024-12-24

**Authors:** Xiameng Lu, Bibo Tan, Liuting Yang, Suning Huang

**Affiliations:** ^1^ Department of Radiation Oncology, Guangxi Medical University Cancer Hospital, Nanning, Guangxi, China; ^2^ Medical College of Oncology, Guangxi Medical University, Nanning, Guangxi, China

**Keywords:** head and neck carcinoma, double primary tumor, immune checkpoint inhibitor, chemotherapy, radiotherapy, case report

## Abstract

The simultaneous occurrence of head and neck squamous carcinoma in two anatomical sites is rare, posing challenges in treatment selection. This paper presents a clinical case of concurrent hypopharyngeal carcinoma and nasopharyngeal carcinoma, successfully treated with a combination of chemoradiotherapy and an immune checkpoint inhibitor. The patient achieved complete remission and progression-free survival of nearly 3 years, with preserved organ function and minimal toxic side effects, leading to a good quality of life. This case highlights the potential of combined concurrent chemoradiotherapy and immune checkpoint inhibitors in managing double primary HNSCC, offering a promising treatment option for these patients.

## Introduction

Hypopharyngeal cancer, a common malignant tumor with a poor prognosis, poses a significant threat to human health and quality of life. Nasopharyngeal cancer typically presents as locally advanced at diagnosis, with radiotherapy being the primary treatment. Although both hypopharyngeal and nasopharyngeal cancers are classified as head and neck squamous carcinoma (HNSCC), the incidence of double primary cancers arising in these two regions is rare. Consequently, there is a scarcity of treatment protocols that specifically address this uncommon condition. Moreover, the potential of immunotherapy in treating double primary cancers warrants further exploration.

In this report, we present the case of a 70-year-old male patient diagnosed with hypopharyngeal and nasopharyngeal cancers who underwent a treatment regimen consisting of chemoradiotherapy and immunotherapy. The patient achieved complete remission in both tumors and no evidence of recurrence or metastasis during 35 months of regular follow-up assessments. Furthermore, the patient experienced minimal toxic side effects and maintained good organ function and a high quality of life.

This case highlights the potential efficacy of combining chemoradiotherapy with immune checkpoint inhibitors (ICIs) managing advanced double primary HNSCC, suggesting a promising treatment approach for patients with similar presentations.

## Case presentation

**Figure 1:**
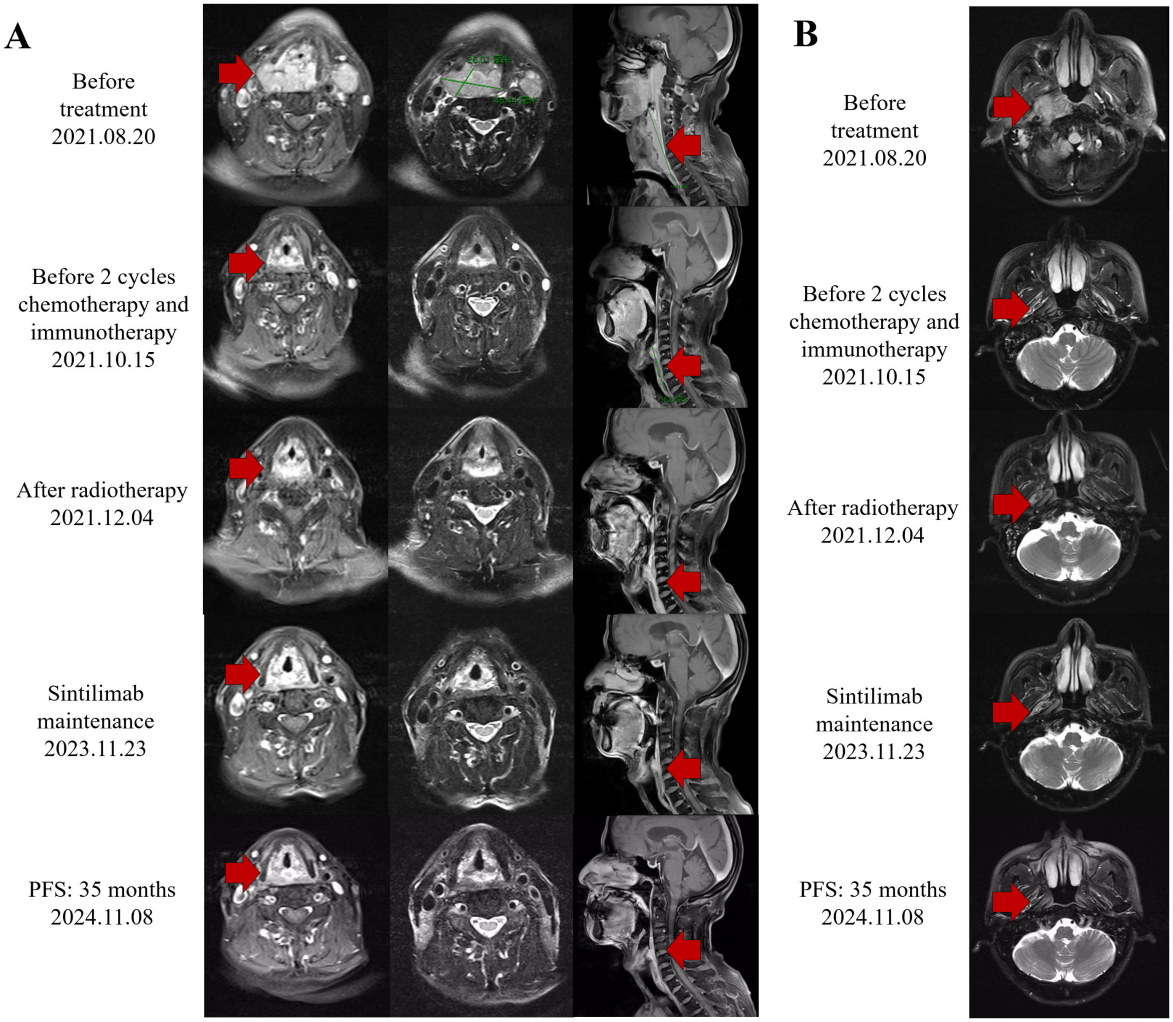
Response evaluation during the clinical course. **(A)** Representative images of the MRI scan revealed the decreasing process of both hypopharyngeal primary cancer and metastasis lymph nodes. Red arrows indicate tumor lesions. **(B)** Representative images of the MRI scan revealed the decreasing process of both nasopharyngeal primary cancer. Red arrows indicate tumor lesions.

**Figure 2:**
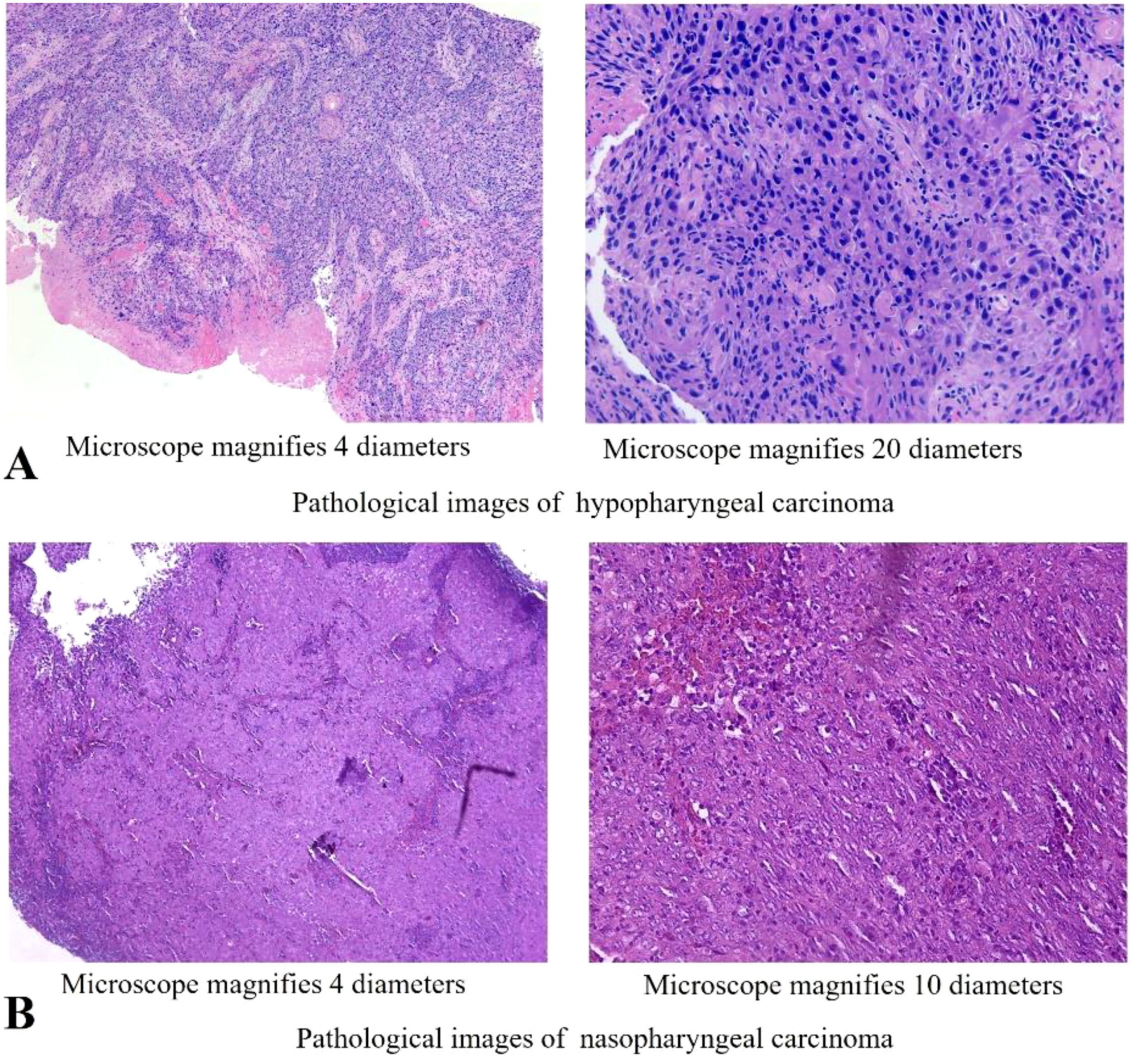
Histopathology of the cancer of this patient. **(A)** Microscopic observation (4x,20x) of H&E staining of the hypopharyngeal lesion. **(B)** Microscopic observation (4x, 10x) of H&E staining of the nasopharyngeal lesion.

We present a case of a 70-year-old male patient. The patient presented with a sore throat and obstruction sensation. Over the past three months, the patient’s throat pain has relapsed repeatedly, with worsening the sensation of swallowing obstruction and difficulty opening the mouth, leading to a consultation at the hospital. A head and neck magnetic resonance imaging (MRI) showed that an abnormal signals in the posterior wall of the hypopharynx, with invasion into the right pyriform sinus, epiglottis, and bilateral aryepiglottic folds. The tumor demonstrated unclear demarcation from the thyroid and compressed the trachea. The nasopharyngeal wall is thickened, with enlarged retropharyngeal lymph nodes (**Figure 1**). PET-CT scans were performed and showed no signs of metastasis in other organs, except for hypermetabolism in the hypopharyngeal, nasopharynx and metastatic lymph nodes in the neck. Subsequently, surgical procedures performed on the patient included direct laryngoscopy, transoral laryngeal mass excision, rigid esophagoscopy, and tracheostomy. The tracheostomy was performed to establish an open airway due to the trachea compression and to prevent potential suffocation during treatment. During the laryngoscopy, a mass was observed on the posterior wall of the hypopharynx, extending into the right pyriform sinus. A biopsy of the mass was taken for pathological diagnosis. The esophagoscopy was performed, and no abnormalities were noted. Pathology indicated keratinized squamous cell carcinoma of the hypopharynx (**Figure 2A**). He then underwent a fibro-laryngoscope examination and revealed a nasopharynx mass. A biopsy of the nasopharyngeal confirmed it was non-keratinizing undifferentiated squamous cell carcinoma (**Figure 2B**), with positive EBERs staining.

The patient was in good physical health prior to treatment, exhibiting no heart, brain, liver, or kidney diseases, and was not on any long-term medication. He worked as a farmer, with no exposure to chemical toxins, industrial dust, or radiation. The patient has a 30-year smoking history, with a smoking index of 450 (30 pack-years). And he has consumed alcohol for 30 years, averaging 50 mg per day. Following his diagnosis in July 2021, he ceased both smoking and alcohol consumption.

In conclusion, the patient presented with a sore throat and odynophagia. Physical examination revealed palpable lymph nodes in the right neck. Imaging examination identified masses in the hypopharynx and the nasopharynx, with local invasion into the thyroid and trachea. Pathological examination indicated different histological type in hypopharynx and nasopharynx. Two anatomical site masses with different histological type raise the certainty of a double primary malignancy. The diagnosis of double primary cancers presents challenges. In terms of hypopharyngeal carcinoma, the tumor was located in the posterior wall of the hypopharynx, invading the pyriform sinus, thyroid and trachea, and was classified as T4a. Bilateral cervical lymph node metastasis leading to a staging of N2c. In terms of NPC staging, the patient exhibited a thickened posterior nasopharyngeal wall without infiltration of the pharyngobasilar fascia, leading to a local classification of T1. Besides, the boundary between the retropharyngeal lymph node and nasopharynx was unclear while the lymph node was anatomically distant from the hypopharyngeal tumor. Therefore, we prefer that nasopharyngeal carcinoma with retropharyngeal lymph node metastasis, leading to a staging of N1. Following the staging criteria for double primary tumors, the diagnosis was as follows: 1. Hypopharyngeal squamous cell carcinoma (T4aN2cM0 IVA, AJCC^8th^); 2. Nasopharyngeal non-keratinizing undifferentiated carcinoma (T1N1M0 II, AJCC^8th^).

**Figure 3:**
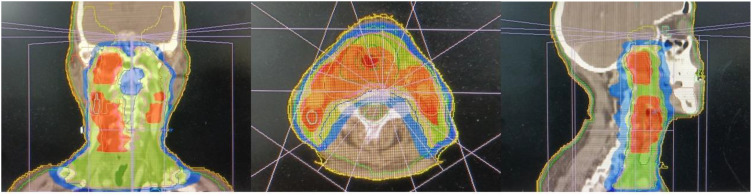
Dose profile of the primary cancers and the metastasis lymph nodes.

Following a multidisciplinary discussion, the treatment approach of “radiotherapy-chemotherapy-immunotherapy” has been established. The patient received three cycles of induction therapy, including albumin-bound paclitaxel combined with cisplatin chemotherapy, along with the ICI sintilimab. The doses were as follows: albumin-bound paclitaxel 260mg/m^2^ d1, cisplatin 75mg/m^2^ d1-2, sintilimab 200mg d1. Subsequently, the patient received concurrent chemotherapy and intensified modulated radiotherapy (IMRT). The gross tumor volume (GTV) included tumors in the hypopharynx and nasopharynx, retropharyngeal lymph nodes, and adjacent organs invaded by the tumor. The GTVnd was defined as the metastatic cervical lymph nodes. The GTV was expanded by 1 cm to establish the clinical tumor volume (CTV), which encompassed potential sites of tumor invasion and the drainage area of the bilateral cervical lymph nodes. The prescribed radiation doses were as follows: 6MeV, X-ray, PGTV: 60.2Gy/28f (2.15Gy/f), PGTVnd-L/R: 60.2Gy/28f (2.15Gy/f), PCTV: 50.4Gy/28f (1.8Gy/f), with acceptable dose limits for normal organs (**Figure 3**). The patient also received the fourth cycle of chemotherapy combined with sintilimab shortly before completing radiotherapy, maintaining the exact dosages as previously mentioned. Throughout the treatment, this patient experienced grade 2 leukopenia, grade 2 acute radiation-induced mucositis, and mild gastrointestinal reactions. A nutrition nursing unit was established in the radiotherapy ward to provide comprehensive nutritional guidance, including intravenous nutrition and oral supplementation. The patient maintained a satisfactory nutritional status, experiencing only a 2 kg weight loss, and therefore did not undergo a gastrostomy.

**Figure 4:**
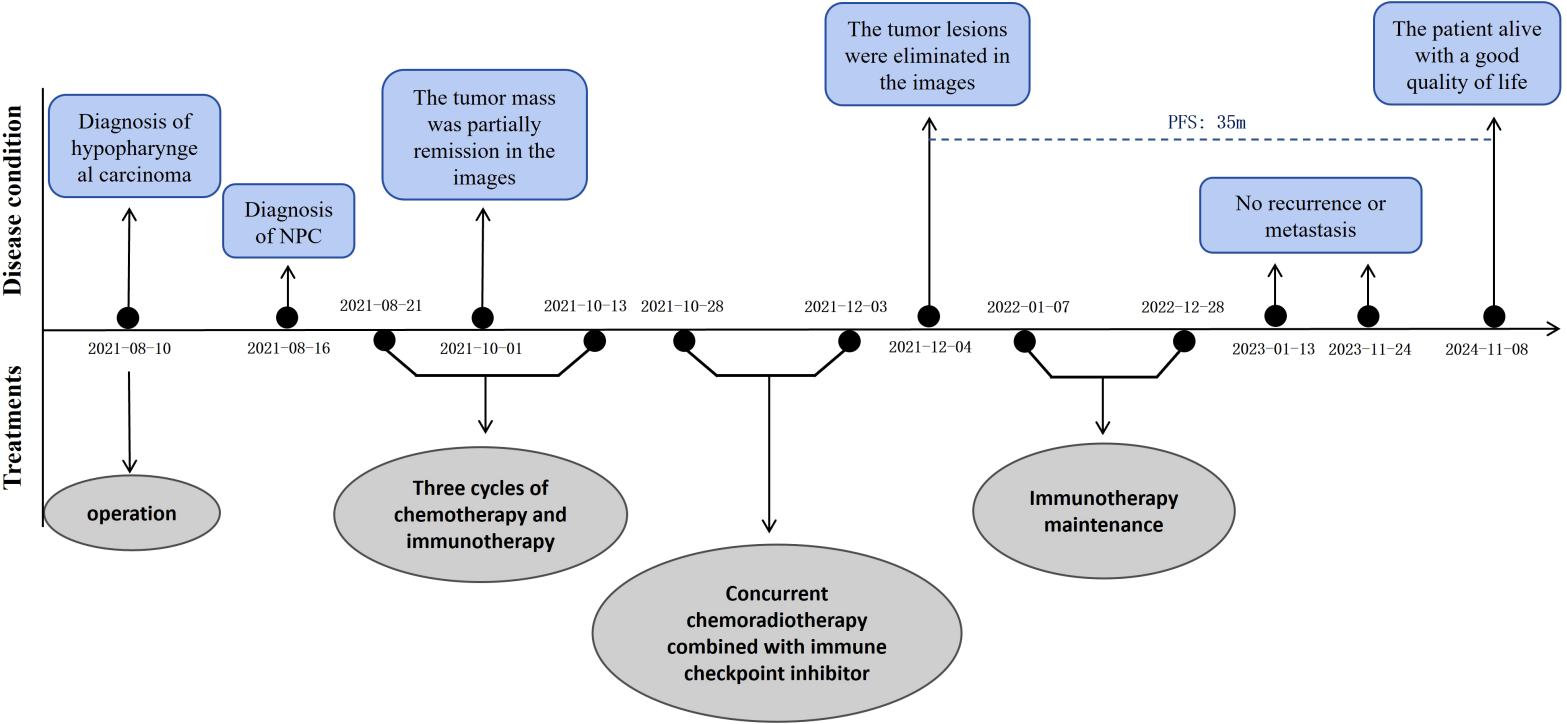
The whole clinical timeline of the patient, with major treatment and disease status. PFS, progression-free survival.

An MRI re-assessment after comprehensive therapy indicated a complete tumor regression, including hypopharyngeal and nasopharyngeal mass, as well as the adjacent peripheral soft tissue and metastatic lymph nodes (**Figure 2**). The nasopharyngoscope confirmed that the tumor mass had complete remission. The patient subsequently received regular immunotherapy maintenance for a year. The therapeutic efficacy was assessed to have achieved a complete clinical response (cCR). Over a 35months follow-up period, the patient’s self-care abilities and organ functions remained usual, with no adverse events or complications reported. The clinical timeline is shown in **Figure 4**.

## Discussion

Multiple primary malignant tumors, referring to the patient simultaneously occurring two or more primary malignant tumor, present significant clinical challenges. The pathogenesis of multiple primary tumors has not been clarified but may be related to genetic susceptibility, viral infection, and radiation induction (1). The double primary head and neck cancer in hypopharyngeal and nasopharyngeal is rare and presents a poor prognosis and low quality of life, posing substantial challenges for clinical treatment.

This case involves a patient with double primary HNSCC. Based on the multiple primary tumors treatment guideline in China and the poorer prognosis associated with hypopharyngeal carcinoma, we prioritized the control of hypopharyngeal cancer while incorporating treatment strategies for NPC (2). The treatment approach of “radiotherapy-chemotherapy-immunotherapy” has been established. For one thing, induction chemotherapy followed by concurrent chemoradiotherapy is recommended by National Comprehensive Cancer Network (NCCN) guideline due to the complicated anatomy in nasopharynx and hypopharynx. Specifically, paclitaxel combined with platinum is suitable for both induction and concurrent chemotherapy in hypopharyngeal carcinoma (category 1). Besides, locally advanced HNSCC presents challenges in effectively targeting cells at the center of the tumor, resulting in treatment resistance. Immunotherapy enhances body’s anti-tumor immunity, enabling the elimination of these resistance tumor cells.

We finalized a treatment consisting of a induction chemotherapy and partially hypofractionated radiotherapy dose of 60.20 Gy over 28 fractions (2.15 Gy per fraction) with concurrent ICI and chemotherapy. On the one side, in the 2022 NCCN guidelines, the combination of paclitaxel with cisplatin was recommended for induction chemotherapy in HNSCC (category 1). Albumin-bound paclitaxel, approved by the US FDA for the first-line treatment of non-small cell lung cancer (3), breast cancer (4), ovarian cancer (5), and pancreatic cancer (6), has been demonstrated to simplify pre-treatments and reduce allergic events during chemotherapy. The dosage was settled: albumin-bound paclitaxel 260mg/m^2^ d1, cisplatin 75mg/m^2^ d1-2. Furthermore, the 2022 NCCN guidelines also recommend paclitaxel and cisplatin as concurrent chemotherapy agents (category 2B) or weekly cisplatin for concurrent chemotherapy following induction chemotherapy (category 2B). Therefore, the patient received three cycles of albumin-bound paclitaxel with cisplatin before radiation and exhibiting a favorable response (**Figure 2**). Then he received the fourth cycle of albumin-bound paclitaxel with cisplatin based on the tumor response and the guideline.

On the other side, China was in the challenging stage of COVID-19 epidemic prevention and control measures at the time of treatment. Hypofractionated radiotherapy, which shortens the overall treatment duration and minimizes patient hospital exposure, was anticipated to lower the risk of COVID-19 infection (7). Evidence indicates that the hypofractionated regimen (2.2-2.75 Gy per fraction, daily for five days a week) demonstrates enhanced efficacy and reduced toxicity in elderly patients with HNSCC (8, 9). The ASTRO and ESTRO guidelines also recommend increasing conventional radiation doses for HNSCC during the pandemic (10). This hypofractionated radiotherapy protocol was determined with the patient’s full informed consent.

Despite receiving chemoradiotherapy, the prognostic survival of patients with local advanced HNSCC, especially double primary carcinoma, remains unsatisfactory. Studies have confirmed that the median survival time for double primary cancers is 22.8 months (1). Researches showed that immunotherapy improved patients’ long-term survival with locally advanced HNSCC. For immunotherapy, pembrolizumab was recommended as a first-line treatment for unresectable HNSCC. Both sintilimab and pembrolizumab are PD-1 inhibitors that limit tumor evasion and activate the immune response. Sintilimab, an IgG4 monoclonal antibody, mitigates tumor-induced immunosuppression by binding to programmed cell death protein 1 (PD-1), inhibiting its interaction with programmed death ligands^2^. Additionally, sintilimab is economically accessible for patients. Research has consistently shown the antitumor efficacy of sintilimab. In recent years, there has been a deep exploration of programmed cell death receptor 1 (PD-1)/programmed death ligand 1 (PD-L1) immune checkpoint inhibitors. Theoretically, ICIs reduce immune evasion by tumor cells and enhance the function of tumor-specific T-cells, potentially improving overall anti-tumor effectiveness (11). Prospective trials have shown that ICIs significantly improve objective response rate (ORR) and 2-year overall survival (OS) in HNSCC patients. A Phase II clinical trial conducted by Professor Zhou and his colleagues investigated the efficacy and safety of sintilimab in patients with platinum-resistant HNSCC. The trial results demonstrated that sintilimab significantly prolonged progression-free survival (PFS) without increasing the incidence of adverse effects (12). Besides, the KEYNOTE-048 study (NCT02358031) at Yale Cancer Center showed that pembrolizumab combined with chemotherapy had a better OS than the standard treatment in HNSCC patients (13). Checkmate 141 and Keynote-040 studies demonstrated a significant OS benefit with the second line of the immune checkpoint inhibitor PD-1 over standard therapy in recurrent or metastatic HNSCC (14, 15). These research findings indicated that sintilimab can be a promising immune checkpoint inhibitor in locally advanced HNSCC, which offering new treatment options for HNSCC or other carcinoma, thereby expanding the therapeutic possibilities for cancer patients.

Furthermore, the optimal duration of maintenance immunotherapy remains uncertain. Based on previous research findings, a maintenance immunotherapy period of 1 to 2 years is a viable option (16, 17). The patient ultimately received ICI maintenance therapy for 1 year but discontinued it for personal reasons. Clinical trials (NCT04557020, NCT04453826, NCT03700476) are currently enrolling participants, aiming to advance medical applications and guide optimal treatment strategies for HNSCC in the era of immunotherapy.

However, there are limitations to the treatments and dilemmas in strategizing the approach. On the one hand, despite the patient achieving nearly complete tumor regression during induction treatment, as well as the bioequivalent dose of the radiotherapy regimen exceeding 70 Gy, conventional fractionation remains the predominant method for treating HNSCC. Some hypofractionated radiotherapy protocols are still in clinical trials, lacking randomized controlled trials and long-term follow-up data. Nevertheless, upon reviewing the available data, many experts have endorsed partially hypofractionated radiotherapy as the preferred dosage during the pandemic, as it minimizes hospital exposure, particularly for elderly patients, and reduces the risk of COVID-19 infection while maintaining effective tumor control without significantly increasing adverse effects (8, 9). On the other hand, immune checkpoint inhibitors may lead to immune-related adverse events (irAE). Therefore, monitoring key indicators regarding the patient’s heart, liver, kidneys, lungs, thyroid, and other organs is essential throughout the treatment and follow-up. This approach enables prompt identification and management of irAE, facilitating timely interventions or drug discontinuation when necessary.

In all, the treatment strategy takes into account the COVID-19 epidemic and the patient’s characteristics while concerning the treatment guidelines. The patient was well-informed about the treatment strategy and demonstrated good compliance, facilitating the treatment process and minimizing side effects. All therapeutic interventions were communicated clearly, and comprehensive informed consent was secured from the patient. After treatment, he regularly received comprehensive examinations, revealing no disfunction in organs and no signs of tumor progression. The patient’s quality of life was satisfactory.

## Conclusion

This patient diagnosed with double primary cancer underwent treatment with sintilimab and chemoradiotherapy, resulting in complete regression of the tumor. This case underscores the efficacy and safety of this regimen for hypopharyngeal carcinoma and nasopharyngeal carcinoma. Nevertheless, further meta-analyses and real-world clinical trials are warranted to validate its efficacy in managing multiple primary HNSCC.

## Data Availability

The original contributions presented in the study are included in the article/Supplementary Material. Further inquiries can be directed to the corresponding author.
